# To What Extent Does Cardiovascular Risk Classification of Patients with Type 2 Diabetes Differ between European Guidelines from 2023, 2021, and 2019? A Cross-Sectional Study

**DOI:** 10.3390/medicina60020334

**Published:** 2024-02-16

**Authors:** Silvia Ana Luca, Raluca Malina Bungau, Sandra Lazar, Ovidiu Potre, Bogdan Timar

**Affiliations:** 1 Department of Cardiology, “Victor Babes” University of Medicine and Pharmacy, 300041 Timisoara, Romania; silvia.luca@umft.ro; 2 Centre for Molecular Research in Nephrology and Vascular Diseases, “Victor Babes” University of Medicine and Pharmacy, 300041 Timisoara, Romania; sandra.lazar@umft.ro (S.L.); bogdan.timar@umft.ro (B.T.); 3 Department of Diabetes, “Pius Brinzeu” Emergency Hospital, 300736 Timisoara, Romania; malina.bungau@umft.ro; 4 First Department of Internal Medicine, “Victor Babes” University of Medicine and Pharmacy, 300041 Timisoara, Romania; 5 Multidisciplinary Research Centre for Malignant Hematological Diseases (CCMHM), “Victor Babes” University of Medicine and Pharmacy, 300041 Timisoara, Romania; 6 Second Department of Internal Medicine, “Victor Babes” University of Medicine and Pharmacy, 300041 Timisoara, Romania

**Keywords:** cardiovascular risk, Type 2 Diabetes, SCORE2-Diabetes, Romanian population

## Abstract

*Background and Objectives*: Type 2 Diabetes (T2DM) is intricately associated with an increased cardiovascular (CV) risk, highlighting the imperative for tailored intervention in the prevention and management of CV diseases. To assess the CV risk and subsequent interventions in patients with diabetes, the European Society of Cardiology (ESC) has been consistently developing and updating specific guidelines for risk assessment and patient management since 2019. The 2023 risk classification method has significantly changed, introducing a novel probability-based assessment through the implementation of SCORE2-Diabetes instrument. This marks a shift from the risk factor-based classification employed in the 2019 and 2021 methods, representing an innovative approach in risk assessment for individuals with T2DM. This study aims to evaluate the differences in the CV risk classification among hospitalized patients with T2DM using the three proposed methods within the Romanian population, a European population considered to be at very high cardiovascular risk. *Materials and Methods*: in a consecutive-case, population-based study design, 70 patients hospitalized with T2DM from a European population characterized by very high CV risk were assessed for CV risk using the three proposed methods. The differences between these classifications were subsequently analyzed. *Results*: In the study group, according to 2023 classification, one patient (1.4%) was classified with moderate CV risk, eight (11.4%) with high cardiovascular risk, and sixty-one (87.2%) with very high cardiovascular risk. A total of 36 patients (51.4%) were classified differently compared to 2021 criteria, the differences being statistically significant (*p* = 0.047), while 13 (18.6%) were different compared to 2019 criteria, the differences being statistically non-significant (*p* = 0.731). By comparing the 2021 to the 2019 ESC Guidelines recommendations, 40 patients had a one-step decrease in cardiovascular risk category, from very high to high risk. *Conclusions*: Most patients included in the analysis were classified as very high CV risk (87.2%). Within a European population characterized by very high CV risk, the SCORE2-Diabetes instrument proves to be a valuable tool, contributing to most step-ups in CV risk classes within the 2023 classification. In a very-high-risk demographic, the 2023 algorithm resulted in different classifications in contrast to the 2021 method but similar classifications observed with the 2019 method.

## 1. Introduction

### 1.1. Background

Patients with Type 2 Diabetes (T2DM) face a significantly increased risk of developing cardiovascular disease (CVD) during their lifetime, manifesting as coronary artery disease (CAD), heart failure, atrial fibrillation, stroke, or as peripheral artery disease [1].

Managing T2DM requires a multifaceted approach that includes not only lifestyle changes and glycemic control but also reducing the overall cardiovascular risk through guided interventions based on cardiovascular (CV) risk factors [2] and the use of glucose-lowering agents with proven CV benefits [3], such as SGLT2 inhibitors [4] and GLP-1 receptor agonists [5].

Considering that CVD represents a leading cause of morbidity and mortality among these patients [6], with a major impact on their prognosis, assessment of cardiovascular risk in individuals with T2DM exhibits some particular aspects [7], previously highlighted in the *2019 ESC Guidelines on Diabetes, Prediabetes and Cardiovascular Disease* [8].

The results of several large cardiovascular and renal outcome trials in patients with diabetes [9] have changed the perspective on the management of CV risk, with two major revisions in 2021 [10] and 2023 [11].

Until 2023, cardiovascular risk stratification in patients with T2DM primarily relied on well-defined, simple-to-assess criteria like age, smoking status, hypertension, and cholesterol levels [12], without taking into consideration individual and specific diabetes-related data, such as age of diagnosis or hemoglobin A1c (HbA1c) levels. The 2023 ESC Guidelines introduce, as an element of novelty, a new prediction model tailored for individuals with T2DM, SCORE2-Diabetes [13]. This new algorithm is developed to estimate the 10-year risk of fatal or non-fatal CV events in individuals with T2DM without atherosclerotic cardiovascular disease (ASCVD) or severe target organ damage (TOD).

In current guidelines, patients with T2DM and clinically established ASCVD or severe TOD are considered to have a very high CV risk. Regarding those individuals without ASCVD or TOD and aged over 40, it is recommended to estimate the 10-year CV risk using the SCORE2-Diabetes algorithm [11].

SCORE2-Diabetes was developed as an extension of the original SCORE2 algorithms used in the general population (without diabetes) [14], using predictors such as age, sex, smoking, systolic blood pressure, total, and HDL cholesterol levels with additional diabetes-related data (age at diagnosis and HbA1c levels) and biomarkers of kidney function [15]. Because CV risk is not homogenously distributed across Europe’s population [16,17], risk prediction models are statistically adjusted to account for clinically relevant differences in CVD rates among various European regions [18], based on World Health Organization cardiovascular mortality rates: low-, moderate-, high-, and very-high-risk regions [19].

The 2023 ESC Guidelines bring an improved CVD risk prediction model for patients with T2DM [13], that accurately reflects the substantial geographical variation in CVD prevalence across Europe, but which requires collecting and entering specific data into an algorithm, an often time-consuming method that is sometimes difficult to utilize in everyday medical practice.

The method of evaluating CV risk in individuals with T2DM has significantly changed in the ESC Guidelines from 2019 to 2023 [12,20]. The main purpose of the study is to evaluate any differences in clustering T2DM patients into CV risk categories using the 2023, 2021, and 2019 ESC Guidelines recommendations, considering the increased workload needed to evaluate the risk using the 2023 criteria. This approach helps against underestimating the individual CV risk, by ensuring that patients in the very-high-risk category benefit from timely interventions like the use of glucose-lowering therapy with proven CV benefits or a more ambitious LDL cholesterol target.

### 1.2. Aims

The study aims to assess the distribution of CV risk in patients with T2DM, in a real-life, consecutive-case scenario, for patients admitted due to metabolic imbalance, based on the updated ESC 2023 Guidelines [11]. Additionally, we aimed to investigate the differences in CV risk classification by comparing the 2023, 2021, and 2019 ESC Guideline recommendations as well as to evaluate the factors that might contribute to a different clustering between these editions.

## 2. Materials and Methods

### 2.1. Study Design

In this non-interventional, consecutive-case, population-based, cross-sectional, single-center study, 70 patients admitted to the Diabetes Clinic of the “Pius Brinzeu” Emergency Hospital Timisoara for metabolic imbalances were enrolled. Data used in the study were collected from patient’s medical records. All data were obtained according to the hospital’s standard of care for patients with T2DM. All patients included in the study provided informed consent for data collection and secondary use of medical data for research purpose. The collected data were used to cluster the enrolled cohort in sub-categories according to the ESC 2023, 2021, and 2019 criteria, respectively. The study protocol was approved by the Local Ethics Committee for Scientifical Research of “Pius Brinzeu” Emergency Hospital Timisoara, approval number 418 from 2023.

### 2.2. Anthropometric, Clinical, and Laboratory Assessments

To cluster patients with T2DM in CV risk categories, certain laboratory data were collected: total cholesterol, triglycerides (TG), high-density lipoprotein cholesterol (HDL-C), LDL-C, and hemoglobin A1c levels. To assess the renal function, eGFR and urinary albumin-creatinine ratio were calculated. Blood pressure values, smoking status, duration of diabetes, and body mass index (BMI) were also determined.

Age (men ≥ 45 years, women ≥ 55 years), hypertension (BP of ≥140/90 mm Hg or use of an antihypertensive drug), current smoking, dyslipidemia, and obesity (BMI of ≥30 kg/m^2^) are considered to be major CV risk factors. Dyslipidemia was defined as total cholesterol of ≥200 mg/dL, TG ≥ 150 mg/dL, LDL-C ≥ 100 mg/dL, HDL-C < 40 mg/dL (men) or <50 mg/dL (women,) or the use of lipid-lowering agents. All data were obtained from patient’s medical records, according to the hospital’s standard of care for patients with T2DM.

### 2.3. Cardiovascular Risk Factorks

Patients were classified into low, moderate, high, and very high CV risk categories according to the ESC Guidelines: in 2019, moderate risk patients are considered those with T2DM < 50 years of age, with DM duration of less than 10 years, and without any additional risk factors. At high risk are patients with DM duration of ≥10 years without TOD plus any other additional CV risk factor. The very-high-risk category includes patients with established CVD, TOD (defined as proteinuria, an eGFR < 30 mL/min/1.73 m^2^, retinopathy, or left ventricular hypertrophy), or three or more major CV risk factors (age, hypertension, smoking, obesity, or dyslipidemia) [8]. The 2021 guidelines included in the moderate-risk category patients with well-controlled DM (<10 years), while high-risk patients were considered those not fulfilling the moderate-risk criteria. As for the very high CV risk category, the 2021 guidelines classify them as having the following: DM patients with established ASCVD or TOD defined as eGFR < 45 mL/min/1.73 m^2^ regardless of albuminuria, or eGFR 45–59 mL/min/1.73 m^2^ and urine albumin–creatinine (UAC) level of 30–300 mg/g, or proteinuria (defined as an UAC level of >300 mg/g), or the presence of microvascular disease in a minimum of three distinct locations (neuropathy, retinopathy, and microalbuminuria) [10]. The 2023 guidelines, through the introduction of the SCORE2-Diabetes algorithm, cluster T2DM patients according to their individual CV risk: a SCORE2-Diabetes of <5% defines patients as low CV risk, 5 to <10% as moderate CV risk, and <20% as high CV risk. As for very high CV risk, it includes T2DM patients with clinically established ASCVD, severe TOD, or a SCORE2-Diabetes value of ≥20% [11].

### 2.4. Patients

The study was conducted among individuals with T2DM aged 40 to 69 years, admitted for metabolic imbalances to the Diabetes Clinic of the “Pius Brinzeu” Emergency Hospital, Timisoara, Romania, between June and October 2023. The cohort consisted of 70 patients diagnosed with T2DM, with or without established ASCVD or severe TOD. Documented clinical ASCVD includes angina pectoralis, history of acute myocardial infarction (MI), acute coronary syndrome (ACS), arterial revascularization, stroke and peripheral artery disease (PAD), and clearly established ASCVD evidenced through imaging, like plaques visualized on a coronary angiography, carotid ultrasound, or CT-angiography. The definition of severe TOD has changed from the ESC Guidelines 2019 to 2021. Initially, in 2019, proteinuria, an eGFR < 30 mL/min/1.73 m^2^, retinopathy, and left ventricular hypertrophy were considered severe TOD. In the 2021 and 2023 guidelines, renal impairment was assessed using both eGFR and microalbuminuria levels, and severe TOD is described as follows: (i) eGFR < 45 mL/min/1.73 m^2^ regardless of albuminuria; (ii) eGFR 45–59 mL/min/1.73 m^2^ and urine albumin–creatinine (UAC) level of 30–300 mg/g; (iii) proteinuria (defined as an UAC level of >300 mg/g) or the presence of microvascular disease in a minimum of three distinct locations (neuropathy, retinopathy, and microalbuminuria). Patients above the age of 69 or below 40 years and those with type 1 DM were excluded from the present study. Patient’s baseline characteristics are presented in Table 1.

### 2.5. Statistical Analysis

Data were collected and analyzed using the Statistical Package for Social Sciences v.27 (IBM Corp. Armonk, NY, USA) and are presented as categorical and relative frequencies (data stored in categorical variables), median and interquartile distances (ordinal variables and numerical variables with non-parametric distributions), and mean and standard deviations (numerical variables with Gaussian distribution). Unpaired *t*-Student’s and ANOVA tests (Gaussian variables), Mann–Whitney U, Kruskal–Wallis, and Wilcoxon signed rank tests (non-parametric variables), and Chi-square or Fisher’s exact tests (categorical variables) were used to assess the significance of differences between or among groups.

In this study, a *p*-value lower than 0.05 is considered the threshold for statistical significance. For the aim of the study, the cohort’s size was dimensioned for a statistical power of 0.80 and 0.95 confidence level.

## 3. Results

In the study group, according to 2023 classification, one patient (1.4%) was classified with moderate cardiovascular risk, eight (11.4%) with high risk, and 61 (87.2%) with very high cardiovascular risk.

**Figure 1:**
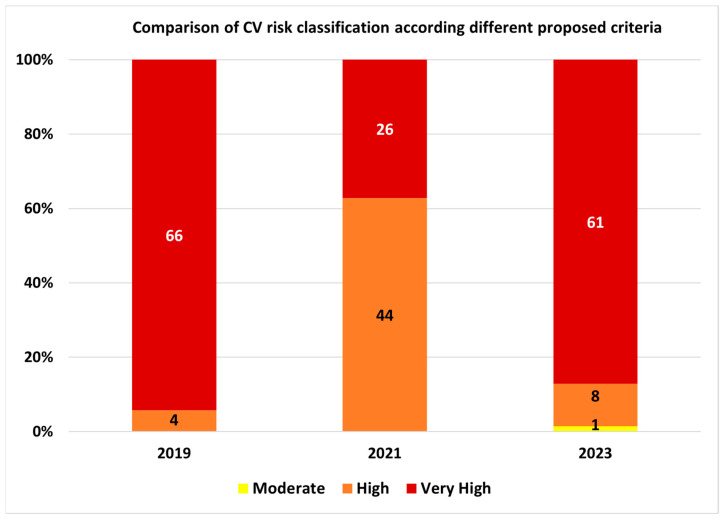
Comparison of CV risk classification between the proposed criteria.

A total of 36 patients (51.4%) were classified differently compared to 2021 criteria and 13 (18.6%) were classified differently compared to 2019 criteria (Figure 1), respectively.

**Figure 2:**
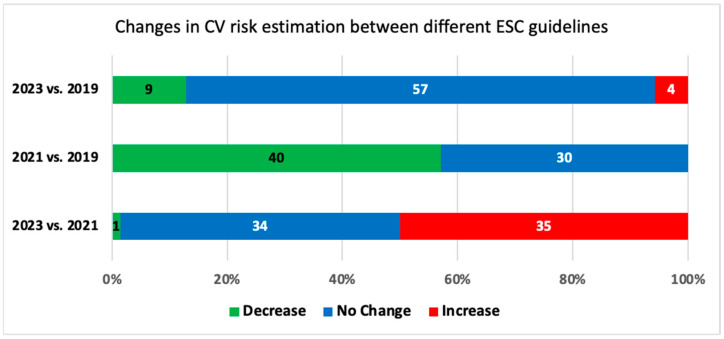
Variation in cardiovascular risk estimates according to different ESC Guidelines.

Regarding the differences in classification between 2023 vs. 2021 criteria, one patient (1.4%) had a one-step decrease in cardiovascular risk category (from high to moderate risk), 35 patients (50.0%) had a one-step increase in cardiovascular risk (from high to very high risk), while 34 patients (48.6%) were classified in the same cardiovascular risk category. When compared to 2019 criteria, one patient (1.4%) had, according to 2023 criteria, a two-step decrease in cardiovascular risk class (from very high to moderate risk), eight patients (11.4%) a one-step decrease (from very high to high risk), and four patients (5.7%) a one-step increase (from high to very high risk), while 57 patients (81.4%) were classified in the same cardiovascular risk category (Figure 2).

**Figure 3:**
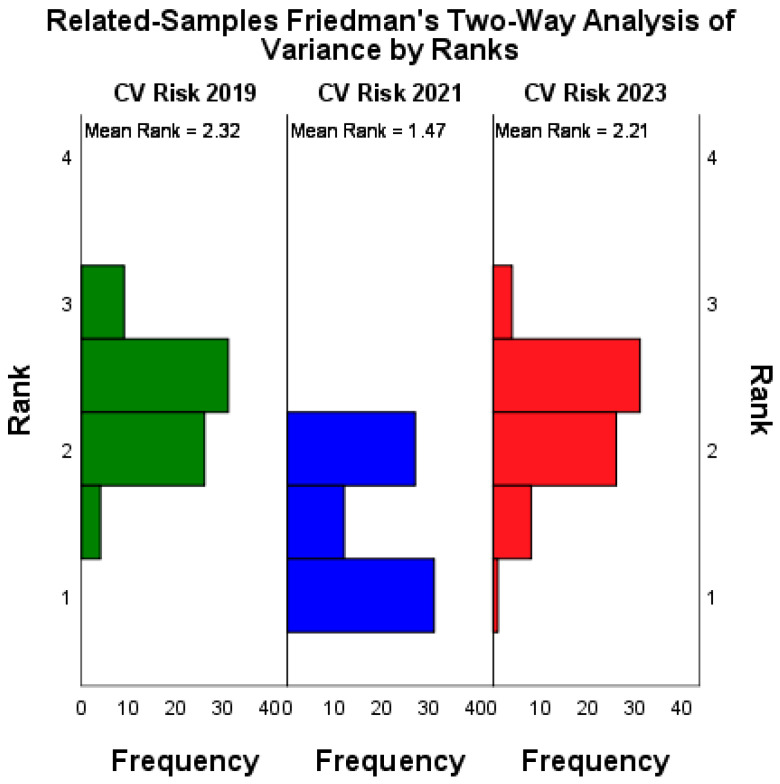
Rank variations (Friedman’s method) of cardiovascular risk categories according to the three analyzed classifications.

The differences in patients’ classification according to the three cardiovascular risk assessment methods analyzed had significant variations (*p* < 0.001; Friedman’s two-way analysis of variance; Figure 3).

Post hoc, paired analysis differences were observed between the ranks of the 2023 vs. 2021 (*p* < 0.001; paired Wilcoxon’s ranks sum test) and 2021 vs. 2019 (*p* < 0.001; paired Wilcoxon’s ranks sum test) classifications, while no statistically significant differences were observed between 2023 vs. 2019 classifications (*p* = 0.499; paired Wilcoxon’s ranks sum test). The detailed results of the post hoc analysis are presented in Table 2.

**Figure 4:**
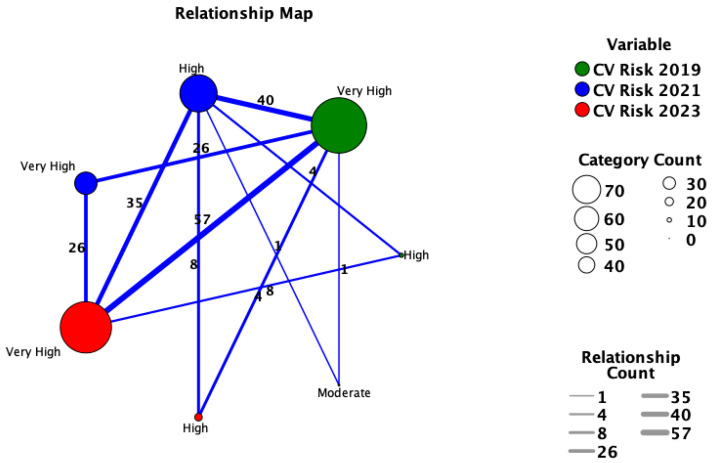
Network map for relationship between the three analyzed cardiovascular risk classifications.

The categorical classification regarding cardiovascular risk had significant differences between the 2023 vs. 2021 method (*p* = 0.047; Pearson’s chi-square test), while no significant differences were observed between 2023 vs. 2019 classification (*p* = 0.731; Pearson’s chi-square test). The relationship matrix between the classifications according to the three methods is presented in Table 3 and the relationship diagram analysis in Figure 4.

In the 2023 cardiovascular risk classification, among the 61 patients (87.1%) included in very high cardiovascular risk, 38 (62.3%) were classified based on the presence of atherosclerotic cardiovascular disease, 22 (36.0%) were classified based on a SCORE2-Diabetes higher than 20%, while one patient (1.7%) was classified based on the presence of severe target organ damage criteria, without the presence of ASCVD. It is noteworthy mentioning that two patients had severe target organ damage associated with the presence of ASCVD.

In the subgroup of patients with ASCVD (thirty-eight patients), regarding the components of ASCVD, twenty patients (52.6%) had coronary artery disease, twelve (31.6%) peripheral artery disease, five (13.2%) a history of myocardial infarction, and three (7.9%) a history of stroke.

**Figure 5:**
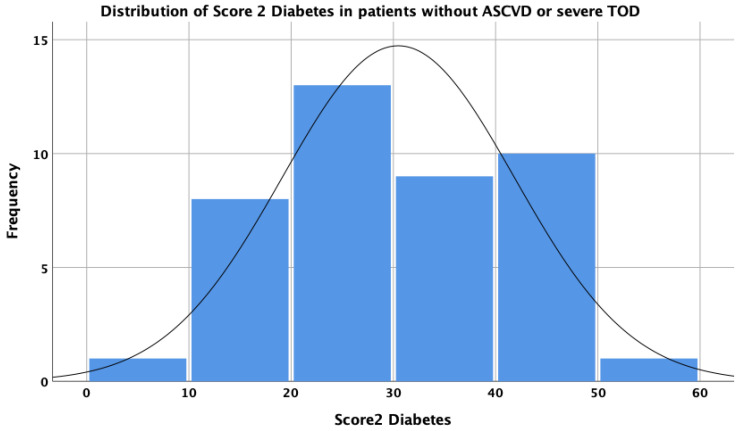
Histogram of SCORE2-Diabetes distribution.

**Figure 6:**
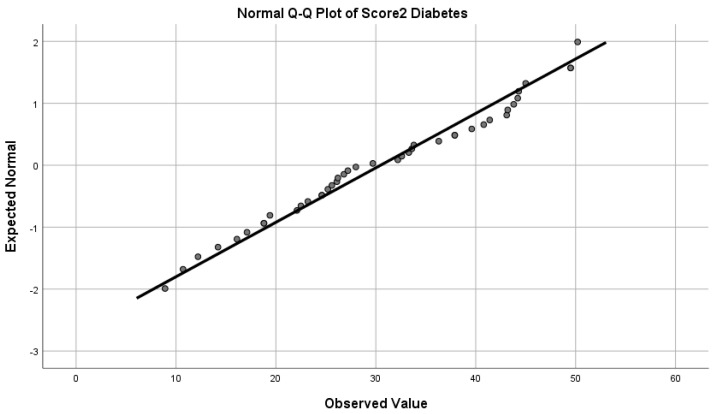
Quantile–quantile plot comparing the SCORE2−Diabetes points to ideal Gaussian distribution.

The distribution of the SCORE2-Diabetes results was quasi-Gaussian (skewness = −0.002; *p* = 0.279; Shapiro–Wilk test; Figure 5), thus demonstrating a good population reproducibility of the SCORE2-Diabetes instrument in a very-high-risk European population of patients with Type 2 Diabetes, with a low Q–Q deviation from normal (Figure 6). Excluding the patients with very high cardiovascular risk due to the presence of ASCVD or severe TOD, in which the assessment of SCORE2-Diabetes was not applicable, in this cohort a median 28.5 points in SCORE2-Diabetes was observed with an interquartile distance of 18.5 points (minimum 8.9 points; maximum 50.2 points).

## 4. Discussion

The study aimed to assess the differences in classifying 70 patients with T2DM hospitalized in Romania into cardiovascular risk classes based on the recommendations of the 2023, 2021, and 2019 ESC Guidelines [8,10,11]. The 2021 risk assessment method for patients with diabetes significantly underestimated the CV risk, with half of the consecutive case enrolled hospitalized patients classified in an inferior risk category versus the 2019 and the 2023 method. The 2023 SCORE2-Diabetes instrument proved to be a valuable tool, with most of the patients that were classified in an increased CV risk category being stepped-up based on the estimation of SCORE2-Diabetes 10-year cardiovascular event probability. Most patients included in the analysis were classified as very high CV risk (87.2%), 11.4% as high, and 1.4% as moderate CV risk, respectively. In very high CV risk European regions, the 2023 classification method was similar to the 2019 method, despite the former method making use just of risk factors and not probability calculations.

On a related note, it could be hypothesized that the 2019 method might overestimate the risk in European low- and moderate-risk regions, where the 2021 approach may be more suitable. Consequently, we can conclude that the added value of the 2023 method, despite its complexity in implementation and calculation, lies in providing a much better estimate, irrespective of the geographical region. This brings a more balanced approach to risk estimation, aligning closely with the 2019 method for high-risk regions and with the 2021 method for low- and moderate-risk regions.

### 4.1. Interpretation of Findings

Compared to the 2023 and 2019 classifications, the ESC Guidelines recommendations from 2021 may have underestimated the cardiovascular risk in the cohort. This discrepancy could be attributed to the fact that, compared to the other guidelines, the 2021 edition, while focusing on adding more criteria to identify patients with severe target organ disease using kidney function measurements (eGFR levels and microalbuminuria) [21,22] or the presence of microvascular disease in at least three different sites [23], omitted to evaluate the impact of additional risk factors such as smoking, obesity, dyslipidemia, hypertension, or age [24]. In contrast, the 2019 guidelines specified that the presence of three or more risk factors automatically classified the patient at a very high cardiovascular risk. Similarly, the 2023 guidelines incorporate all these risk factors using the innovative SCORE2-Diabetes algorithm, which takes into account parameters such as age, eGFR levels, smoking status, systolic blood pressure values, cholesterol levels, and HbA1c [13].

While all the methods above evaluate the 10-year risk of patients with T2DM to develop fatal or non-fatal CV disease in a similar manner, no significant differences seem to have been found between the 2019 and 2023 methods, as compared to the 2021 method. All three methods classify very-high-risk groups as patients with established CV disease, but the definition of severe target organ damage (TOD) has changed over the years. In 2019, patients with an eGFR < 30 mL/min/1.73 m^2^, proteinuria, retinopathy, or LV hypertrophy were considered to have severe TOD. From 2021, this definition changed to include eGFR levels < 45 mL/min/1.73 m^2^, irrespective of the albumin–creatinine ratio, eGFR 45–59 mL/min/1.73 m^2^ with an ACR between 30–300 mg/g, proteinuria (ACR > 300 mg/g), and the presence of microvascular disease in at least three different areas. As mentioned earlier, in the 2019 guidelines, patients with three or more CV risk factors were classified as being at very high risk. Similarly, in 2023, the value of the individual SCORE2-Diabetes can cluster individuals into CV risk categories, and patients with a SCORE2-Diabetes ≥ 20% are considered to be at very high CV risk. The assessment of CV risk factors may be the reason why the 2019 and 2023 methods are very similar at clustering our cohort into CV risk categories [25].

Based on our estimations, the 2019 and 2023 guidelines’ criteria lack significant differences in categorizing T2DM patients in European high and very-high-risk regions, with both methods providing an accurate estimation of the overall 10-year CV risk in these populations. However, as demonstrated above, the 2021 guidelines’ criteria tend to underestimate the risk in these regions, while for moderate and low-risk regions this method may overestimate the risk.

### 4.2. Contextualization within The Existing Literature

To the best of our knowledge, this is the first study to assess variations in CV risk stratification among patients with T2DM obtained via consecutive-case enrollment, using the 2019, 2021, and 2023 ESC Guidelines, conducted in a very-high-risk European region in terms of CVD mortality rates.

A study conducted on the Renal Insufficiency And Cardiovascular Events (RIACE) Italian Multicenter Study cohort compared how the sample was clustered into the 2019 and 2021 ESC risk categories, while also examining the risk of all-cause mortality within each of these categories [26]. The conclusion was that less than 1% of participants fell in the moderate-risk category. Furthermore, under the 2019 classification, one-third of the participants were classified as high-risk, and two-thirds as very-high-risk, while the 2021 classification showed a reversal in these proportions. The shift was attributed to the reallocation of patients with three or more additional ASCVD risk factors from the very-high-risk to the high-risk category in the 2021 ESC Guidelines. It should be reminded that Italy is considered a moderate-risk region in terms of CV mortality rates.

In assessing prognostic performance, the 2019 ESC/EASD risk stratification model demonstrated inferiority when compared to both SCORE and single NT-proBNP assessment in predicting 10-year, all-cause and CV death in patients with T2DM [27].

Data from a large cross-sectional study conducted in the Mediterranean region of Spain [28], a low-risk European region, classified T2DM patients according to the 2019 ESC Guidelines into low-, moderate-, high- and very-high-risk categories. Findings suggest that at least half of the cohort is at very high CV risk and more than a third of those without established CVD displayed very-high-risk of developing CVD.

In a large observational, retrospective study conducted in Italy, Pintaudi et al. [29] examined clinical profiles among patients with T2DM. Their findings, according to the 2019 ESC Guidelines criteria, revealed that 78.5% of patients with T2DM included in the analysis were at very high risk, 20.9% at high risk, and only 0.6% at moderate risk. Within the moderate-risk category, individuals demonstrated a lower mean age and a relatively short duration of diabetes; among those identified as high risk, nearly half had a diabetes duration of more than 10 years. Most individuals classified as having very high risk had three or more cardiovascular risk factors.

The results of similar studies that used the 2019 ESC Guideline CV risk stratification [26,28,29] show that most patients included in the analysis were at very high CV risk, findings very similar to the results of our study (64 out of 70 patients were at very high risk according to the 2019 method).

Our study was conducted on patients with T2DM in Romania, a very-high-risk European region according to ESC [30]. When assessing the CV risk, it is crucial to note the importance of the patient’s demographics [31], a factor that can modify the overall CV risk in individuals with T2DM. As an element of novelty, the 2023 guidelines, through the implementation of the SCORE2-Diabetes algorithm which accounts for regional differences alongside conventional risk factors, enhance the accuracy of CV risk estimation in this population already at an elevated risk for adverse cardiovascular outcomes [32].

The cohort included in the analysis was obtained in a consecutive-case enrollment and included 70 patients with T2DM originating from a country considered to be a very-high-risk region. All individuals were hospitalized for metabolic imbalances, resulting in a left-skewed distribution of patients’ risk due to a higher prevalence of additional risk factors compared to non-hospitalized individuals with T2DM [33].

### 4.3. Implications for Clinical Practice

Clinicians need a simple yet precise method to evaluate the risk of their patients. One particular category needing special attention concerning cardiovascular adverse outcomes are patients with T2DM. The importance of accurate CV risk assessment in diabetic patients is crucial and should be conducted regularly, especially in countries like Romania and similar high-risk regions [34].

In alignment with the ESC Guidelines, LDL cholesterol targets vary across different CV risk categories [35]. By applying the 2021 method, many patients did not have ambitious LDLc targets as intended, potentially exposing them to an additional risk of developing cardiovascular events due to higher exposure to elevated levels of LDLc [36].

The 2023 ESC Guidelines suggest, with a class I recommendation, that individuals with T2DM identified as being at a very high risk should initiate treatment with glucose-lowering medication with proven CV benefits, such as SGLT-2 inhibitors and/or GLP-1 receptor agonists, independent of HbA1c levels [11].

By using the 2019 or 2023 method, a comprehensive and accurate risk stratification is established for patients originating from high-risk regions. This approach helps against underestimating the individual CV risk, by ensuring that patients are in the recommended LDLc targets and by facilitating timely application of targeted interventions for improved patient outcomes [37]. Furthermore, it provides the opportunity of initiating suitable glucose-lowering therapies with proven CV benefits where necessary [38], addressing the specific needs of individuals from high-risk regions by focusing on optimizing CV outcomes.

### 4.4. Limitations

Our study has several limitations. Firstly, it was conducted in a cohort including exclusively inpatients. Hospitalized patients may exhibit an increased risk compared to outpatients due to the presence of additional comorbidities or risk factors [39]. Secondly, all participants included in our research are from Romania, a very-high-risk European region in terms of CV mortality [40]. The demographic composition of our cohort can significantly influence the distribution of patients across risk categories [31], thereby potentially increasing the overall CV risk for individuals originating from very-high-risk regions. Thirdly, the sample size was estimated to achieve a confidence level of 0.95 with a statistical power of 0.80, ensuring optimal reproducibility of results. However, it is important to note that an increased sample size might elevate the probability of a type 1 statistical error, potentially resulting in decreased confidence if the results were to be positive.

### 4.5. Future Research Directions

In this non-interventional, consecutive-case, population-based, cross-sectional, single-center study, 70 patients with T2DM hospitalized in Romania, a very-high-risk European region in terms of CVD mortality rates [40], were included.

Inpatients often have multiple comorbidities or more severe medical conditions than outpatients [39], which can lead to findings that may not be applicable to individuals with less severe forms of the condition, less comorbidities, or those managed in outpatient settings. The demographic characteristics of inpatients may not accurately reflect the diversity of the overall population by being skewed towards certain age groups or socioeconomic statuses [31]. This highlights the need for a study conducted with consecutive case enrolment from an outpatient setting in a very-high-risk region to more evenly represent the targeted population and extend the applicability of its findings to a broader audience.

Demographics can significantly contribute to variations in CV risk, particularly visible in patients coming from a very-high-risk region like Romania, where the susceptibility to CVD is elevated [40]. The interesting aspect lies in understanding the extent to which the geographical region influences the classification of CV risk among individuals from low-, moderate-, or high-risk European regions. This prompts the necessity of conducting parallel studies to appreciate the impact of regional differences on CV risk stratification.

### 4.6. Concluding Remarks

In essence, the evolving landscape of CV risk assessment and management emphasizes the demand for a nuanced approach [41] that not only considers established risk factors but also is feasible and time-efficient in practice, especially in populations at heightened risk, such as those with T2DM in high-risk regions like Romania. Notable differences were found between the 2023 and 2021 method, suggesting that the 2021 guidelines may underestimate the CV risk in very-high-risk European regions. However, similarities between the 2023 and 2019 classification indicate that the 2019 method is as accurate as, but simpler and more practical than, the 2023 one. The 2019 method may overestimate the risk in European low- and moderate-risk regions, where the 2021 approach may be more suitable. Despite its complexity, the 2023 method proves beneficial, aligning with the 2019 method for very-high- and high-risk regions and the 2021 method for low- and moderate-risk regions. Employing either the 2019 or 2023 methods ensures comprehensive and accurate risk stratification for patients from very-high-risk regions and guards against underestimating individual CV risk, ensuring that patients meet recommended LDLc targets and facilitating timely interventions for enhanced patient outcomes [39]. Furthermore, it offers the opportunity to initiate suitable glucose-lowering therapies with proven CV benefits as needed [3], addressing the specific needs of individuals from very-high-risk regions and focusing on optimizing CV outcomes.

## 5. Conclusions

In very high CV risk populations from Europe, the 2021 risk assessment method for patients with diabetes significantly underestimated the CV risk, with half of the consecutive-case, enrolled, hospitalized patients being classified in an inferior risk category versus the 2023 method. By comparing the 2021 to the 2019 ESC Guidelines recommendations, 40 patients had a one-step decrease in cardiovascular risk category, from very high to high risk. In hospitalized patients with diabetes from very high CV risk regions, according to the 2023 classification and the SCORE2-Diabetes instrument, most patients included in the analysis were classified as very high CV risk (87.2%), 11.4% as high, and 1.4% as moderate CV risk, respectively. In very high CV risk European regions, the 2023 classification method was similar to the 2019 method, despite the former method making use just of risk factors and not probability calculations.

In very high CV risk European regions, the 2023 classification method was similar to the 2019 method, despite the former method making use just of risk factors and not probability calculations.

## Figures and Tables

**Table 1:** Patient’s baseline characteristics.

Parameter	Median	Interquartile Range
Age (years)	62	54–65
Diabetes Duration (Years)	9	4–16
BMI (kg/m^2^)	30.0	25.3–35.2
HbA1c (percentage points)	8.2	7.0–9.9

**Table 2:** Post hoc analysis for the pairwise significance comparison in cardiovascular risk category variations between the three classifications.

Post Hoc Pairwise Comparisons			
	Test Statistic	Std. Test Statistic	*p*
CV Risk 2021 vs. CV Risk 2023	−0.736	−4.353	<0.001
CV Risk 2021 vs. CV Risk 2019	0.850	5.029	<0.001
CV Risk 2023 vs. CV Risk 2019	0.114	0.676	0.499

Each row tests the null hypothesis that the Sample 1 and Sample 2 distributions are the same. Asymptotic significances (2-sided tests) are displayed. The significance level is 0.05. Significance values have been adjusted using the Bonferroni correction for multiple tests.

**Table 3:** Relationship matrix regarding patient’s classification among the three analyzed criteria.

			CV Risk 2023			
			Moderate	High	Very High	*p*-Value
CV Risk 2021	High	Count	1	8	35	0.047
		% within CV Risk 2023	100.0%	100.0%	57.4%	
	Very High	Count	0	0	26	
		% within CV Risk 2023	0.0%	0.0%	42.6%	
CV Risk 2019	High	Count	0	0	4	0.731
		% within CV Risk 2023	0.0%	0.0%	6.6%	
	Very High	Count	1	8	57	
		% within CV Risk 2023	100.0%	100.0%	93.4%	

## Data Availability

The data presented in this study are available on request from the corresponding author. The data are not publicly available due to the hospital’s privacy policy.
